# Cepstral Peak Prominence Values for Clinical Voice Evaluation

**DOI:** 10.1044/2020_AJSLP-20-00001

**Published:** 2020-07-13

**Authors:** Olivia Murton, Robert Hillman, Daryush Mehta

**Affiliations:** a Speech and Hearing Bioscience and Technology, Division of Medical Sciences, Harvard Medical School, Boston, MA; b Center for Laryngeal Surgery and Voice Rehabilitation, Massachusetts General Hospital, Boston; c MGH Institute of Health Professions, Boston, MA; d Department of Surgery, Harvard Medical School, Boston, MA

## Abstract

**Purpose:**

The goal of this study was to employ frequently used analysis methods and tasks to identify values for cepstral peak prominence (CPP) that can aid clinical voice evaluation. Experiment 1 identified CPP values to distinguish speakers with and without voice disorders. Experiment 2 was an initial attempt to estimate auditory-perceptual ratings of overall dysphonia severity using CPP values.

**Method:**

CPP was computed using the Analysis of Dysphonia in Speech and Voice (ADSV) program and Praat. Experiment 1 included recordings from 295 patients with medically diagnosed voice disorders and 50 vocally healthy control speakers. Speakers produced sustained /a/ vowels and the English language Rainbow Passage. CPP cutoff values that best distinguished patient and control speakers were identified. Experiment 2 analyzed recordings from 32 English speakers with varying dysphonia severity and provided preliminary validation of the Experiment 1 cutoffs. Speakers sustained the /a/ vowel and read four sentences from the Consensus Auditory-Perceptual Evaluation of Voice protocol. Trained listeners provided auditory-perceptual ratings of overall dysphonia for the recordings, which were estimated using CPP values in a linear regression model whose performance was evaluated using the coefficient of determination (*r* ^2^).

**Results:**

Experiment 1 identified CPP cutoff values of 11.46 dB (ADSV) and 14.45 dB (Praat) for the sustained /a/ vowels and 6.11 dB (ADSV) and 9.33 dB (Praat) for the Rainbow Passage. CPP values below those thresholds indicated the presence of a voice disorder with up to 94.5% accuracy. In Experiment 2, CPP values estimated ratings of overall dysphonia with *r* ^2^ values up to .74.

**Conclusions:**

The CPP cutoff values identified in Experiment 1 provide normative reference points for clinical voice evaluation based on sustained /a/ vowels and the Rainbow Passage. Experiment 2 provides an initial predictive framework that can be used to relate CPP values to the auditory perception of overall dysphonia severity based on sustained /a/ vowels and Consensus Auditory-Perceptual Evaluation of Voice sentences.

Recent work in acoustic voice analysis has increasingly supported the cepstral peak prominence (CPP) as an objective measure of breathiness and overall dysphonia. In 2018, guidance from the American Speech-Language-Hearing Association (ASHA) recommended CPP as a tool for “measuring the overall level of noise in the vocal signal” and as “a general measure of dysphonia” (Patel et al., 2018). In this recommendation, CPP replaces previous measures of acoustic perturbation, including jitter, shimmer, and harmonics-to-noise ratio. Those traditional measures can only be extracted from sustained vowels and rely on fundamental frequency computation, which may not be reliable for voices with more than moderate dysphonia. In contrast, CPP can be extracted from connected speech and sustained vowels and does not require direct computation of the fundamental frequency.

A growing body of work has demonstrated CPP's ability to differentiate perceptually dysphonic and nondysphonic voices across languages, disorder types, and speaking tasks. Many of these findings in English speakers are reviewed by Fraile and Godino-Llorente (2014), which also provides an overview of the algorithms underlying CPP computation. Other work has also examined CPP in languages other than English, including Spanish (Delgado-Hernández et al., 2019; Núñez-Batalla et al., 2019), Korean (Lee et al., 2019; Yu et al., 2018), and Turkish (Aydinli et al., 2019). In general, these studies find that lower CPP values are well correlated with increases in dysphonia severity based on auditory-perceptual judgments.

The clinical use of CPP is currently limited by a lack of objective guidelines that specify when values are likely to indicate abnormality. Such guidelines would increase the potential for CPP to function as a screening measure (i.e., probability of a voice disorder being present) and make it easier for clinicians to meaningfully interpret CPP, particularly with respect to treatment-related changes (i.e., helping determine whether posttreatment vocal function and/or voice quality more closely approximate normal). Ideally, such guidelines would be based on the analysis methods and tasks that are most frequently used for clinical voice evaluation and include cutoff values/thresholds for detecting the presence or absence of a voice disorder, as well as information about how CPP values relate to dysphonia severity.

## CPP Conceptualization

The cepstrum typically used in voice and speech analysis is given by the inverse Fourier transform of the acoustic spectrum. This process can be intuitively understood as a “spectrum of a spectrum.” First, the waveform is Fourier-transformed into the spectral domain. Then, the logarithm of that spectrum is taken, and another (inverse) Fourier transform is performed into the “cepstral” domain (Fraile & Godino-Llorente, 2014; Heman-Ackah et al., 2003). The horizontal axis of a spectrum shows a range of frequencies. By analogy, the horizontal axis of the cepstrum is a time-like dimension termed “quefrency,” an anagram of “frequency,” just as “cepstrum” is an anagram of “spectrum” (Oppenheim & Schafer, 2004). The periodic harmonic peaks in the spectrum are represented as a single large peak (and its harmonics) in the cepstrum around a quefrency corresponding to the period of the voice signal. The height (i.e., “prominence”) of that peak relative to a regression line through the overall cepstrum is called the “cepstral peak prominence” or CPP and is typically reported in units of decibels. CPP values therefore fall into a continuous range, where lower values are typically correlated with greater levels of dysphonia.

**Figure 1.**
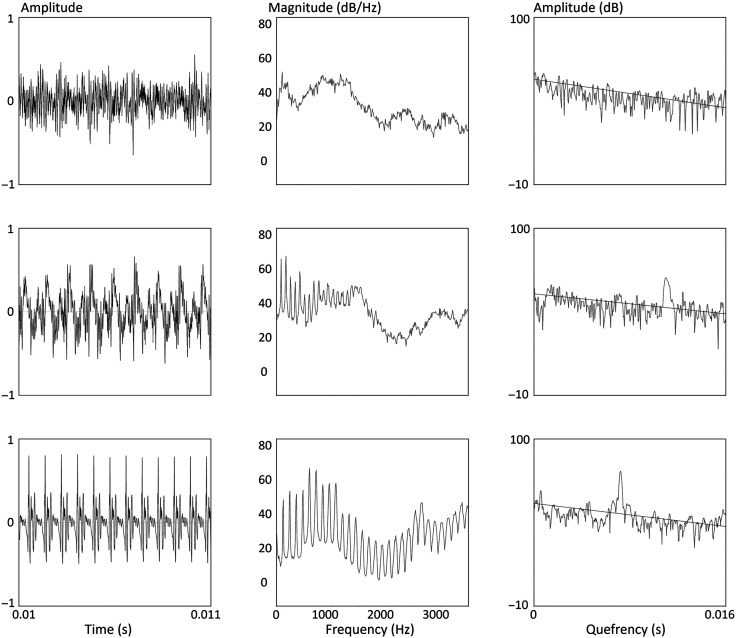
Waveform (left), spectrum (center), and cepstrum (right) from speakers with aphonia (top row), nonaphonic but disordered voice quality (center row), and no voice disorder (bottom row).

Figure 1 illustrates this CPP calculation process for /a/ vowels from three speakers exhibiting a typical, dysphonic, and aphonic voice, respectively. For each row, the leftmost image shows a section of the /a/ vowel's waveform. The center image shows the vowel's spectrum, and the rightmost image shows the vowel's cepstrum and the regression line used in calculating the cepstrum. The vowel from the top row comes from an aphonic speaker, so no harmonics are visible in the spectrum and no peak is apparent in the cepstrum. The vowel in the bottom row comes from a speaker with no voice disorder, so harmonics are prominent in the spectrum and the CPP is well above the regression line. The vowel in the middle row was produced by a nonaphonic speaker with disordered voice quality, so the CPP height is lower relative to that of the typical speaker.

To distinguish normal from disordered voices, the continuous range of CPP values needs to be divided into groups at one or more thresholds. Each potential CPP threshold could yield a different sensitivity (true positive rate [TPR]) and specificity (true negative rate [TNR]). In general, a desirable threshold will have high values for both of these quantities (although it may not always possible to maximize both TPR and TNR at the same time). For any test, a threshold should be chosen such that the rate and type of errors are both acceptable for that test's goal.

## Previous Work

Table 1 summarizes prior studies that examined the ability of CPP to distinguish healthy from pathological voices. In general, they did so by obtaining auditory-perceptual ratings (typically of overall severity and/or breathiness) and correlating those perceptual values with objective CPP values. They often also established CPP cutoff thresholds by dividing the perceptual ratings into two or more categories of dysphonia severity and determining CPP's performance in classifying voices into those groups.

**Table 1.** Summary of previous work identifying clinically relevant cepstral peak prominence (CPP) cutoff values.

Author	Year	Language	Study size	CPP method	Group classification	Sustained vowel CPP cutoff	Running speech CPP cutoff
Heman-Ackah et al.	2003	English	281 patients (176F/105M)	CPPS (Hillenbrand)	Perceptually mild vs. severe dysphonia	10 dB	5 dB
Heman-Ackah et al.	2014	English	835 patients, 50 controls	CPPS (Hillenbrand)	Perceptually normal vs. dysphonic	n/a	4.0 dB
Yu et al.	2018	Korean	214 patients (142F/72M), 74 controls (47F/27M)	ADSV CPP	Perceptually normal vs. dysphonic	12 dB	7 dB
Núñez-Batalla et al.	2019	Spanish	72 patients, 52 controls	CPPS (Praat)	Normative values (not cutoff values)	Female: 16.0 dB Male: 16.4 dB	Female: 7.9–11.3 dB Male: 7.8–10.9 dB (cutoff varies with sentence)
Aydinli et al.	2019	Turkish	27 patients, 27 controls (40M/14F, pediatric)	ADSV CPP	Nodules diagnosis vs. normal voices	No thresholds, but found significantly lower CPP in pediatric speakers with nodules vs. age- and sex-matched controls for most, but not all, speaking tasks.	
Delgado-Hernández et al.	2019	Spanish	136 patients, 47 controls	CPPS (Praat) in two configurations	Perceptually normal vs. dysphonic	Configuration 1: 23.62 dB 2: 13.96 dB	Configuration 1: 18.4 dB 2: 8.37 dB
Lee et al.	2019	Korean	1,029 patients (512M/517F)	ADSV CPP	Normal vs. mild	10 dB	7.7 dB
					Mild vs. moderate	7.5 dB	5.4 dB
					Moderate vs. severe	4.1 dB	2.9 dB

*Note.* F = female; M = male; CPPS = smoothed cepstral peak prominence; ADSV = Analysis of Dysphonia in Speech and Voice.

Many of these studies included both sustained vowels and continuous speech tasks. In all of those cases, CPP thresholds were lower for continuous speech tasks compared to sustained vowels. This general finding suggests that it is important to keep speech tasks consistent when comparing CPP values across recordings. Additionally, the choice of algorithm for calculating CPP is critically important, as different algorithms produce values in different ranges. The three major CPP computation algorithms in these studies are Hillenbrand and Houde's (1996) algorithm for calculating smoothed CPP (CPPS), Praat's CPPS algorithm (Boersma & Weenink, 2018), and the CPP computation method in the Analysis of Dysphonia in Speech and Voice (ADSV; Version 3.4.2, PENTAX Medical).

Two studies by Heman-Ackah et al. (2003, 2014) used Hillenbrand and Houde's (1996) algorithm to identify CPPS thresholds that distinguish voices with severe dysphonia from voices with mild or no dysphonia. These studies provide a basis for comparison of threshold CPP values for sustained vowels and running speech in English speakers. However, because they excluded speakers with moderate dysphonia, it is not clear that those thresholds are appropriate for use in all speakers.

Studies by Núñez-Batalla et al. (2019) and Delgado-Hernández et al. (2019) used Praat's CPPS algorithm to investigate CPP in Spanish speakers with and without diagnoses of voice disorders. Núñez-Batalla et al. identified normative CPPS values by computing the averages and standard deviations of the control groups' CPPS values for each task, rather than identifying threshold values to separate speakers with and without voice disorders. Delgado-Hernández et al. used two distinct configurations of Praat—the default configuration (Configuration 1) and the configuration used to calculate the Acoustic Voice Quality Index (Configuration 2)—to find CPPS cutoff thresholds based on auditory-perceptual ratings of overall severity. However, that result has not been replicated with English language speakers or with other voice analysis programs (e.g., ADSV) that clinicians may also use.

Several studies have used the ADSV program to analyze CPP in Korean (Lee et al., 2019; Yu et al., 2018) and Turkish (Aydinli et al., 2019) speakers. Lee et al. (2019) reported CPP values that distinguished speakers with varying levels of dysphonia, whereas Yu et al. (2018) reported CPP values that separated speakers with and without dysphonia. Aydinli et al. (2019) found lower CPP values in Turkish-speaking children with nodules compared to age- and sex-matched controls but did not report specific CPP thresholds to distinguish those populations.

In a related work, Awan et al. (2016) attempted to find clinically relevant cutoff values for the Cepstral Spectral Index of Dysphonia (CSID), a computational estimate of dysphonia severity that incorporates CPP and measures of spectral energy. They defined three groups of patients with “disordered voices” according to different criteria: (a) “dysphonia-positive” patients according to auditory-perceptual ratings by trained speech-language pathology students, (b) “laryngoscopic-positive” patients based on signs and symptoms visible on laryngeal stroboscopy, and (c) “Voice Handicap Index (VHI)–positive” patients with a value greater than 12 on the 30-item VHI (Jacobson et al., 1997). Awan et al. found that CSID best distinguished dysphonia-positive participants from dysphonia-negative ones. CSID was less accurate for the laryngoscopic and VHI classifications. The VHI classification is arguably the least relevant comparison to CPP, since CPP is not designed to reflect a speaker's self-perception of vocal health/function.

As illustrated by the preceding review, CPP values can vary widely with different speaking tasks and computation algorithms. This variation arises in part because tasks differ in their degree of voicing, and computation algorithms differ in how they treat unvoiced segments. Watts et al. (2017) compared CPP values from Praat and ADSV. English and Flemish speakers produced sustained vowels (/a/) and continuous speech. The Flemish vowel and sentence recordings had correlation coefficients of .93 in ADSV and .88 in Praat, whereas the English vowels and sentences had correlation coefficients of .92 and .96, respectively.

A major difference between these algorithms is ADSV's use of a voicing activity detector. In ADSV, frames with negative CPP (i.e., cepstral peak below the regression line) are not considered for analysis. ADSV's use of the voicing activity detector might explain why the correlation was higher for the English sentence, which was almost fully voiced, than for the Flemish sentence, which contained many unvoiced segments. Unvoiced segments are not typically periodic and are likely to have very low CPP. This result suggests the need to use the same speaking tasks when comparing CPP values from continuous speech, especially when not using a voicing activity detector. Speech samples with different degrees of voicing may yield artificially different CPP values.

## Current Work

In this study, we follow up on the recent ASHA recommendation to use CPP in the clinical assessment of voice (Patel et al., 2018). Unlike previous studies, we use ADSV and Praat to analyze two English language data sets and identify CPP cutoff values to detect probable voice disorders. To our knowledge, no published work has proposed clinically relevant CPP cutoff values for English speakers based on these widely used voice analysis software products. ADSV is a commercially available and supported product widely used by clinicians. Praat is free software available online that is increasingly being used for clinical assessment of voice and speech due to its ease of use, graphical user interface, and scripting features. Hillenbrand and Houde's algorithm has been used for research study and not typically for clinical use—potentially due to lack of support and a user-friendly interface—and therefore is not evaluated in this work.

In Experiment 1, we investigate CPP as a screening tool to predict the presence of a voice disorder using a voice database (Massachusetts Eye and Ear Infirmary [MEEI], 1994) that has been analyzed in many other studies. In Experiment 2, we evaluate the performance of CPP to predict the auditory perception of dysphonia severity using a smaller data set of acoustic recordings that has been rigorously evaluated by trained listeners using ASHA's recommended protocol for the Consensus Auditory-Perceptual Evaluation of Voice (CAPE-V; Kempster et al., 2009). Our goal is to aid practitioners who wish to use the objective measure of CPP as part of their clinical assessment and monitoring of patients with voice disorders.

## Experiment 1: CPP Cutoff Values for Detecting the Potential Presence of a Voice Disorder

## Method

### Database

The MEEI Voice Disorders Database consists of recordings from 687 patients diagnosed with voice disorders and 53 vocally healthy control speakers (MEEI, 1994). The speakers were recorded between 1992 and 1994 at the MEEI's Voice and Speech Lab and Kay Elemetrics (now part of PENTAX Medical). The database consists of sustained /a/ vowel productions from 657 of the patients and Rainbow Passage readings from 662 of the patients. Only 1 s of each sustained vowel and the first 12 s of each Rainbow Passage are available in the database.

In this study, we excluded three control speakers who had a history of smoking. We also excluded a total of 392 patients with the following classifications: Five were classified as “normal,” 51 were classified as postsurgery or posttherapy, 89 were missing a diagnosis, and 247 were classified generically as “pathological voice.” Patients with the generic “pathological voice” classification were excluded because including individuals that lack a definitive/standard diagnosis would introduce uncertainty about the composition and integrity of the pathological data set and make the results less clinically interpretable or applicable. After these exclusions, the database consisted of 295 voice patients and 50 controls. The voice patients' primary diagnoses are presented in Table 2. Table 3 summarizes these speakers' demographics.

**Table 2.** Primary diagnoses of the 295 voice patients in the Massachusetts Eye and Ear Infirmary Voice Disorders Database whose recordings remained in the analysis after exclusion criteria were applied in Experiment 1.

Primary diagnosis	Count
Neurological (92)	
Paralysis/paresis	62
Spasmodic dysphonia	19
Other neurological	11
Muscle tension dysphonia	49
Nodules or polyps	41
Lesions (including cyst, mass, dysplasia)	41
Edema (including Reinke's edema)	41
Scar and/or trauma	15
Presbyphonia	8
Partial laryngectomy	5
Other (arthritis, tuberculosis, laryngocele)	3
**Total**	**295**

**Table 3.** Sex and age distributions of speakers in the Massachusetts Eye and Ear Infirmary Voice Disorders Database analyzed in Experiment 1.

Group	Female	Male	Median age (years)	Age range (years)
Controls	30	20	36.5	22–59
Patients	183	112	45	13–93

All of the speakers in our data set produced both the sustained vowel and the Rainbow Passage, except for four voice patients who produced only the sustained vowel. Therefore, our data set consisted of 345 vowel recordings (295 patients, 50 controls) and 341 Rainbow Passage recordings (291 patients, 50 controls).

### Acoustic and Statistical Analysis

Each recording was analyzed in ADSV (Version 3.4.2) using the program's default settings. The “CPP/EXP Mean (dB)” parameter was extracted to yield CPP for each recording.

Each recording was also analyzed in Praat (Version 6.0.40) using a PowerCepstrogram (60-Hz pitch floor, 2-ms time step, 5-kHz maximum frequency, and pre-emphasis from 50 Hz). CPPS was calculated from each PowerCepstrogram with the following settings: subtract tilt before smoothing = “no”; time averaging window = 0.01 s; quefrency averaging window = 0.001 s; peak search pitch range = 60–330 Hz; tolerance = 0.05; interpolation = “Parabolic”; tilt line quefrency range = 0.001–0 s (no upper bound); line type = “Straight”; fit method = “Robust.” These settings are identical to those used by Watts et al. (2017) and Brockmann-Bauser et al. (2019).

As discussed above, previous studies have found substantially different CPP cutoff thresholds for sustained vowels and continuous speech. Therefore, the sustained vowel and Rainbow Passage recordings were treated separately throughout the analysis. For each task, we identified every CPP value that any participant produced on that task and calculated several performance metrics based on each value. These performance metrics included TPR, TNR, false positive rate (FPR), positive predictive value (PPV), accuracy, and Youden's *J* index, which is given by sensitivity + specificity − 1 (= TPR + TNR − 1). Therefore, Youden's *J* is 1 only when neither false positives nor false negatives are present. It is also not affected by the relative sizes of the positive and negative groups (Youden, 1950). That property is useful for studies in which most people in a study fall into the same class. For example, studies based on people who present to voice clinics are likely to have many more dysphonic voices than controls.

We plotted the series of TPRs against the FPRs to generate a receiver operating characteristic (ROC) curve and calculated the area under the ROC curve to evaluate the overall classification performance. An under the ROC curve closer to 1 indicates better classification performance. The CPP threshold yielding the maximum Youden index was also identified for both the sustained vowels and running speech. This analysis was performed for the ADSV CPP and Praat CPPS calculations separately.

## Results

Table 4 reports the classification performance for discriminating patients versus controls using thresholds for ADSV CPP and Praat CPPS that yielded maximum Youden's *J*.

**Table 4.** Threshold values and performance measures for the Analysis of Dysphonia in Speech and Voice (ADSV)–based cepstral peak prominence (CPP) and Praat-based smoothed CPP (CPPS) classifiers.

Variable	ADSV CPP		Praat CPPS	
	Sustained vowels	Rainbow Passage	Sustained vowels	Rainbow Passage
Threshold	11.46 dB	6.11 dB	14.45 dB	9.33 dB
ROC AUC	.91	.95	.93	.98
Accuracy	79.4%	87.7%	77.4%	94.5%
TPR	0.77	0.87	0.74	0.95
FPR	0.08	0.08	0.02	0.10
TNR	0.92	0.92	0.98	0.90
PPV	0.98	0.98	0.99	0.98
Youden's *J*	0.69	0.79	0.72	0.85

*Note.* ROC = receiver operating characteristic; AUC = under the ROC curve; TPR = true positive rate; FPR = false positive rate; TNR = true negative rate; PPV = positive predictive value.

### ADSV CPP

**Figure 2.**
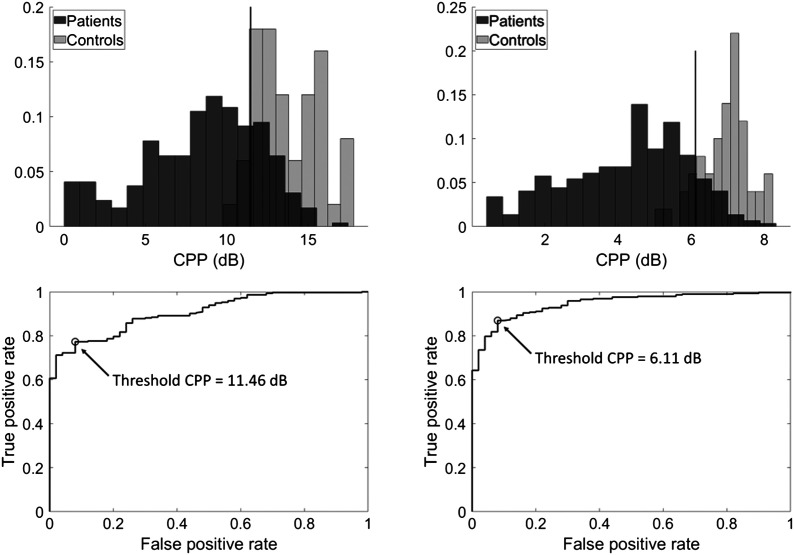
Top row: Histogram of Analysis of Dysphonia in Speech and Voice–based cepstral peak prominence (CPP) values from patients with voice disorders (dark) and vocally healthy individuals (light) for sustained vowels (left) and continuous speech (right). Total bin counts sum to 1 within each group. Vertical lines indicate thresholds derived from the maximum Youden's index. Bottom row: Receiver operating characteristic curves plotting true positive versus false positive rates at various CPP thresholds for sustained vowels (left) and continuous speech (right). The “positive” class is the patient group. Open circles indicate the CPP threshold given by the maximum Youden's index.

Figure 2 (top row) shows the distribution of ADSV-based CPP values in participants with and without voice disorders for the sustained vowel and continuous speech conditions. The histograms are normalized such that the heights of each condition's bars sum to 1. Inspecting these histograms shows that CPPs from controls' and patients' voices typically fall into distinct ranges, with patients' voices showing much wider variation than those of controls. Figure 2 (bottom row) shows the ROC curves for the sustained vowel and continuous speech conditions, with the CPP cutoff value indicating maximum Youden's index labeled on each.

### Praat CPPS

**Figure 3.**
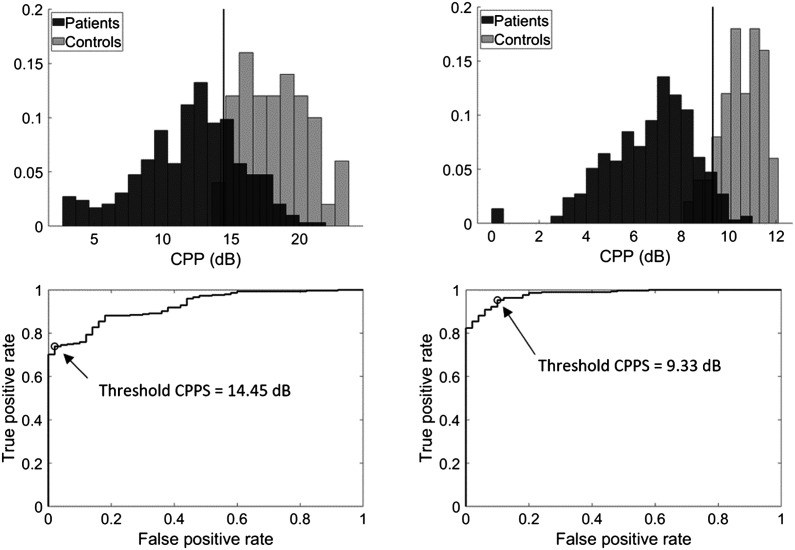
Top row: Histogram of Praat-based smoothed cepstral peak prominence (CPPS) values from patients with voice disorders (dark) and vocally healthy individuals (light) for sustained vowels (left) and continuous speech (right). Total bin counts sum to 1 within each group. Vertical lines indicate thresholds derived from the maximum Youden's index. The “positive” class is the patient group. Bottom row: Receiver operating characteristic curves plotting true positive versus false positive rates at various CPPS thresholds for sustained vowels (left) and continuous speech (right). Open circles indicate the threshold given by the maximum Youden's index.

Figure 3 shows the distributions of Praat-based CPPS values (top row) and ROC curves (bottom row) for the sustained vowel and continuous speech conditions as described for Figure 2. The histograms are normalized such that the heights of each condition's bars sum to 1. Like the ADSV-based CPP values, Praat CPPS separates control and patient voices well, with a wider range of CPPS values for patients' voices than for controls' voices.

## Experiment 2: Estimating Dysphonia Severity Using CPP Values

## Method

### Database

In Experiment 2, we analyzed a data set consisting of 32 speakers that was first published in Awan et al. (2010). This data set includes 24 speakers with voice disorders (12 female and 12 male speakers) and eight speakers with typical voices (four female and four male speakers) based on auditory-perceptual judgment and self-report. The data set also contains auditory-perceptual judgments of voice from 25 trained speech-language pathology graduate student listeners (Kempster et al., 2009). In contrast, the Experiment 1 data set contains only a binary categorization of speakers with and without voice disorder diagnoses. Experiment 2's large set of listener ratings provides a valuable opportunity to quantitatively relate CPP to auditory-perceptual judgments of voice, using a continuous scale rather than the binary decision from Experiment 1. The voice-related diagnoses of the 24 speakers with voice disorders are summarized in Table 5. Each speaker produced a sustained /a/ vowel and four of the six CAPE-V sentences targeting various voicing behaviors: easy onsets (S2: “How hard did he hit him?”), full voicing (S3: “We were away a year ago”), hard glottal attacks (S4: “We eat eggs every Easter”), and voiceless stops (S6: “Peter will keep at the peak”; Kempster et al., 2009).

**Table 5.** Diagnoses of speakers in Experiment 2 (from Awan et al., 2010).

Diagnosis	Count	Male	Female
Paralysis/paresis	8	4	4
Muscle tension dysphonia	4	2	2
Cyst	4	1	3
Nodules/polyp	3	1	2
Papilloma	2	2	0
Amyloidosis	1	1	0
Cancer	1	1	0
Reinke's edema	1	0	1
**Total**	**24**	**12**	**12**

Twenty-five trained speech-language pathology graduate students participated in five separate listening sessions to produce five ratings of each utterance for overall severity, roughness, breathiness, and strain according to the CAPE-V evaluation criteria. These listener ratings resulted in 125 ratings (25 listeners × 5 ratings each) for each sustained vowel and CAPE-V sentence. Measures of inter- and intrarater reliability indicated that the listeners accurately distinguished between dysphonia severity levels and provided highly reliable ratings. The listener rating process is described in more detail in the study of Awan et al. (2010). Final ratings of each dysphonia category were computed as the mean over all 125 ratings for that category. For this study, overall severity was selected as the auditory-perceptual category to be estimated using CPP values.

### Acoustic and Statistical Analysis

Following the same procedure as our analysis of the MEEI corpus, each recording was analyzed in ADSV using the program's default settings. The “CPP/EXP Mean (dB)” parameter was extracted to yield a CPP value for each recording. Additionally, for each recording, all 125 listener ratings of overall severity were averaged to yield a single measure of perceived overall dysphonia severity. Separately, the Praat-based CPPS was calculated using the parameters described in Experiment 1.

Linear regression models were computed using the MATLAB fitlm function to assess CPP's ability to estimate the perceptual ratings of overall severity. These models were computed separately for the vowels and each of the four CAPE-V sentences, resulting in five regression models of the form predicted severity rating = *m* x CPP + *b* . For each model, the coefficient of determination (*r* ^2^) was computed, and 95% prediction intervals (PI_95_) were calculated as follows:

$$PI_{95}=y_{i}\underset{-}{+}t_{\text{crit}}*\text{RMSE}*\sqrt{1+\frac{1}{n}+\frac{{\left(x_{i}-\bar{x}\right)}^{2}}{\sum_{j}{\left(x_{j}-\bar{x}\right)}^{2}}}$$ (1)

where *n* represents the number of observations in the model, *y_i_* represents the model's prediction given *x_i_, t*_crit_ represents the critical *t* value for *n* − 2 observations at a 95% significance level, and RMSE is the root-mean-square error of the regression model.

Finally, the sustained vowels' CPP cutoff scores from Experiment 1 (11.46 dB for ADSV and 14.45 dB for Praat) were used to classify the patient and control speakers in Experiment 2 as an initial of validation of the cutoff scores in an independent data set. TPR, TNR, FPR, PPV, and accuracy were calculated for the vowel-based ADSV and Praat cutoffs separately. Note that FPR and TNR always sum to 1, so if one value is high, then the other will be low.

## Results

### ADSV CPP

**Figure 4.**
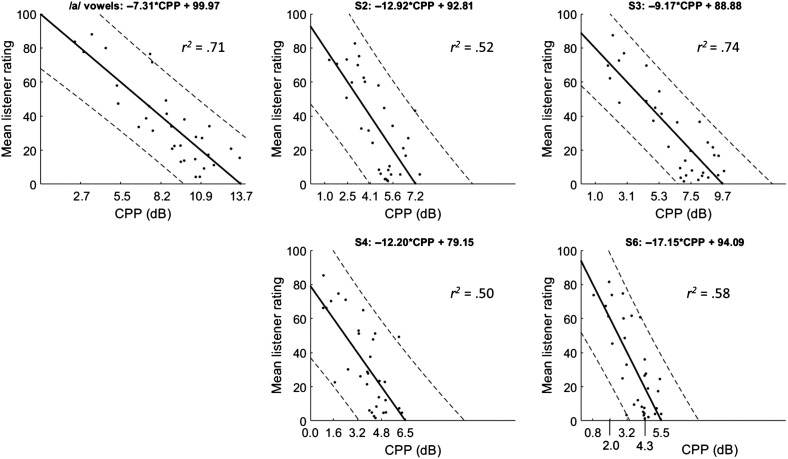
Correlations between Analysis of Dysphonia in Speech and Voice–based cepstral peak prominence (CPP) and listener rating of overall severity for each speaking task. Solid lines indicate best-fit regression line, and dashed lines show 95% prediction intervals. The *x*-axis tick labels show the CPP values corresponding to each *y*-axis tick label based on the regression line.

Figure 4 shows best-fit regression lines and PI_95_ linking ADSV-based CPP to the mean listener rating of overall severity for each task separately. In general, CPP and overall severity were well correlated, with *r* ^2^ ranging from .5 to .74, depending on the task. As expected, the *r* ^2^ value of .71 for the /a/ vowels is very close to the *r* ^2^ of .70 found by Awan et al. (2010) on this data set using a similar version of ADSV. Subfigure titles include the regression line equation relating listener ratings to CPP, and the *r* ^2^ value is indicated in each subfigure. The *x*-axis tick labels show the CPP values that correspond to each *y*-axis label based on the regression model. For example, a CPP value of 2.7 dB on an /a/ vowel corresponds to a mean listener rating of 80 on the CAPE-V overall severity scale (Kempster et al., 2009). Dashed lines indicate PI_95_ for each point on the regression line. The CPP threshold value of 11.46 dB from Experiment 1 yielded an Experiment 2 accuracy of 68.8% (22/32), TPR of 87.5% (21/24), TNR of 12.5% (1/8), PPV of 75% (21/28), and FPR of 87.5% (7/8).

### Praat CPPS

**Figure 5.**
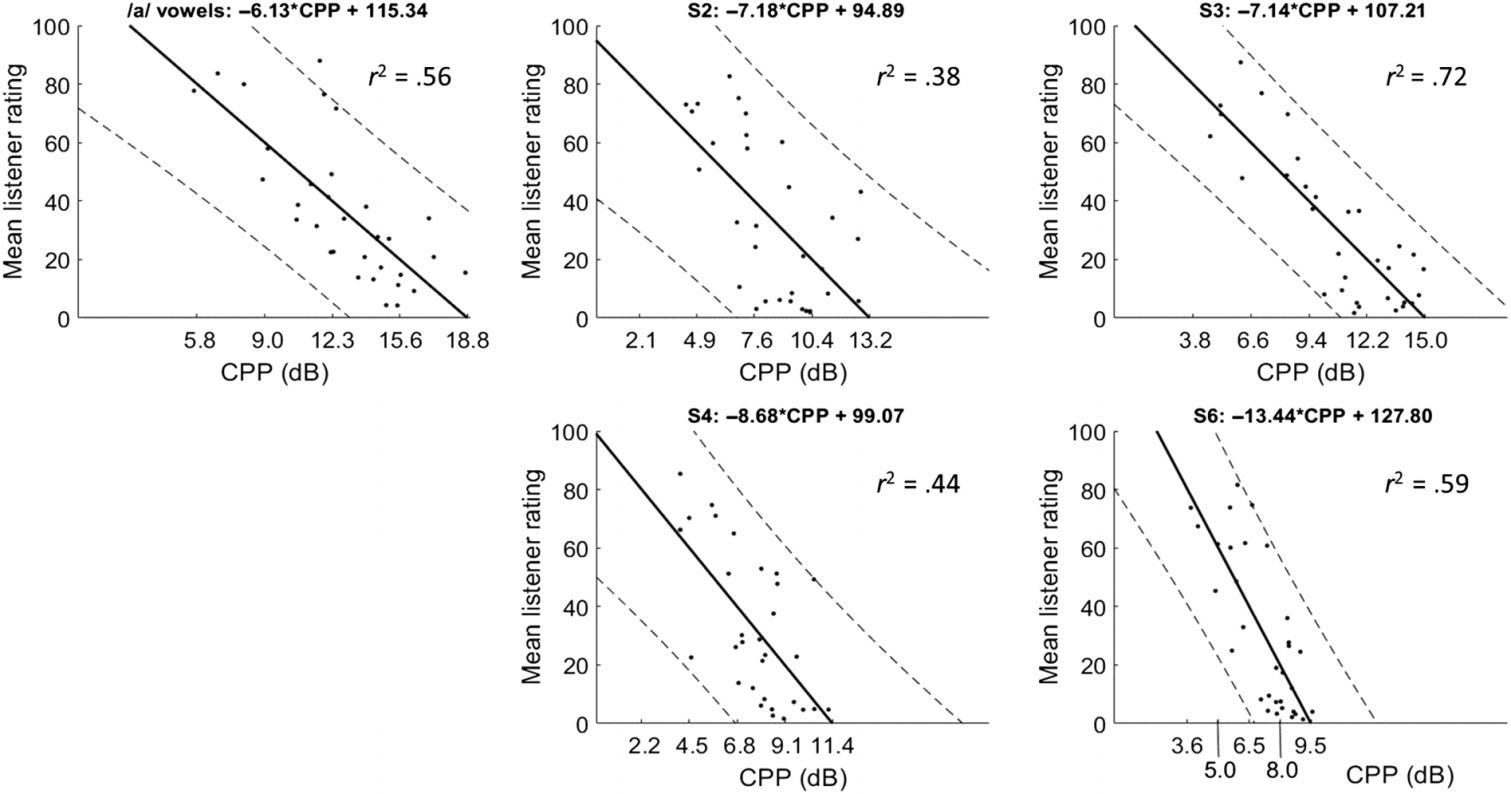
Correlations between Praat smoothed cepstral peak prominence (CPPS) and listener rating of overall severity for each speaking task. Solid lines indicate best-fit regression line, and dashed lines show 95% prediction intervals. The *x*-axis tick labels show the CPPS values corresponding to each *y*-axis tick label based on the regression line.

Figure 5 shows regression lines and PI_95_ relating Praat CPPS to mean listener ratings of overall severity. The figure was generated following the same procedure used to create Figure 4, with the regression line, *r* ^2^ value, and PI_95_ indicated on each subfigure. The *r* ^2^ values relating overall severity to CPPS ranged from .38 to .72. The CPPS threshold value of 14.45 dB from Experiment 1 yielded an Experiment 2 accuracy of 75% (24/32), TPR of 79% (19/24), TNR of 62.5% (5/8), PPV of 86.4% (19/22), and FPR of 37.5% (3/8).

## Discussion

CPP is widely understood to be an accurate predictor of dysphonia severity. To our knowledge, this is the first study to identify CPP values that are based on using both ADSV- and Praat-based analysis methods on the same well-controlled databases of English speakers.

### CPP Cutoff Values and Comparisons to Previous Studies

Our results from Experiment 1 suggest that ADSV-based CPP values below 11.46 dB for sustained vowels and below 6.11 dB for the Rainbow Passage should be considered indicative of a voice disorder. Praat-based CPPS values below 14.45 dB for sustained vowels or below 9.33 dB for continuous speech indicate a high probability of the presence of a voice disorder. The cutoff values indicated here represent only one possible estimate of a CPP clinical cutoff, so values in the near vicinity of the cutoff should be given further consideration when used clinically. There are several other possible methods of determining an appropriate cutoff threshold that could be applied in future work to balance specificity and sensitivity in different ways (Habibzadeh et al., 2016; Unal, 2017).

Separately, our results from Experiment 2 suggest quantitative relationships between CPP values and perceptual ratings of dysphonia severity levels. The regression lines above each plot in Figures 4 and 5 can be used to predict dysphonia severity based on ADSV CPP (see Figure 4) or Praat CPPS (see Figure 5). For example, if a clinician used Praat to analyze a speaker's /a/ value and found a CPPS value of 10 dB, the predicted CAPE-V overall severity rating would be approximately 54: −6.13 x 10 + 115.34 = 54.04. That said, the data set for Experiment 2 is relatively small, and the PI_95_ ranges are fairly large, so these results should be applied with caution and call for additional study with larger databases.

Our results are comparable to those from previous similar studies. For example, Yu et al. (2018) found ADSV-based CPP thresholds of approximately 12 dB for sustained vowels and 7 dB for running speech in Korean speakers. Heman-Ackah et al. (2003) used Hillenbrand's algorithm with English speakers and found that thresholds of 10 dB for sustained vowels and 5 dB for running speech distinguished mild from severe dysphonia. These thresholds are somewhat lower than ours, but our Experiment 1 cutoff values are intended to distinguish patients with voice disorders versus vocally healthy controls instead of mild versus severe dysphonia. Additionally, our Praat CPPS thresholds are similar to the ones identified by Delgado-Hernández et al. (2019), whose Praat “Configuration 2” settings yielded CPP thresholds of 13.96 dB for sustained vowels and 8.37 dB for continuous speech.

#### Validation of Experiment 1 Thresholds Using Experiment 2 Data Set

We used the cutoff values for /a/ vowels from Experiment 1 to classify the patient versus control voices from Experiment 2. We did not perform that validation for the connected speech because the speaking tasks were different (Rainbow Passage vs. CAPE-V sentences) and the resulting CPP values could not be directly compared. The accuracy scores for ADSV-based CPP and Praat-based CPPS were similar, at 68.8% for ADSV and 71.9% for Praat. The TPR was high for both ADSV (21/24) and Praat (18/24), but the TNR was higher for Praat (5/8) than for ADSV (1/8). Although the TNR for ADSV seems low, there were four control speakers whose ADSV-based CPP values were close to the cutoff value (< 1 dB below). The remaining three control speakers were the same ones who were below the Praat CPPS threshold. Those speakers also had the highest overall severity ratings of the control group, as judged by the trained listeners. Overall, these results indicate that the Experiment 1 CPP cutoffs classified most of the Experiment 2 speakers accurately and that clinicians should use particular caution when interpreting CPP values that are close to the clinical cutoff thresholds.

#### Choices of Task and Computation Algorithm Are Important

An important finding from Experiment 2 is the wide variation in CPP values among the different CAPE-V sentence tasks. This result suggests that between and within speaker comparisons of CPP values for continuous speech should be based on the same speech material (e.g., same sentences from the CAPE-V or reading passage). Within a single speech task, speakers are likely to be similar to each other in their production of nonvoiced elements like consonants and pauses, so changes in speakers' vocal quality can be directly observed (Hillenbrand & Houde, 1996).

One possible explanation for the variation in running speech CPP values is the differing amounts of voicing in each CAPE-V sentence. All four sentences tended to have lower CPP values than the sustained vowels. Sentence 3 was fully voiced (“We were away a year ago”) and tended to have the highest CPP values of the four sentences. In contrast, Sentence 6 had many voiceless stops (“Peter will keep at the peak”) and tended to have the lowest CPP values. This pattern also occurs for the Praat-based CPPS values, with Sentence 3 tending to have the highest CPPS and Sentence 6 having the lowest.

These results suggest that unvoiced frames are being included in the ADSV-based CPP and Praat-based CPPS calculations. A sentence with many voiceless consonants, especially stop consonants, is likely to contain many unvoiced frames with very low CPP. Including these frames in a calculation of an utterance's average CPP can artificially lower the overall CPP. Praat's CPPS calculation does not include voicing activity detection, but ADSV's CPP algorithm does. Although ADSV does incorporate voicing detection, our results suggest that ADSV's voice activity detector may not filter out all the unvoiced frames in an utterance. As noted in Awan et al. (2010), incomplete or no voicing detection could cause the observed differences between CPP values for sentences with and without unvoiced segments. Improving voice activity detection could reduce the effects of unvoiced frames on a CPP calculation and facilitate comparison between CPPs of different speech tasks.

The use of voicing detection is particularly complicated for voices with aphonia, particularly those with intermittent aphonia. Frames that do not contain voicing due to aphonia, pausing, voiceless consonants, and so forth yield low CPP values. If voicing detection is inaccurate or not used, those nonvoiced frames will be included in the computation of average CPP, so including aphonic segments will tend to decrease the average CPP. In that case, the low CPP accurately reflects a noise-like or aphonic voice quality that is clinically meaningful. If very accurate voicing detection is applied, however, only voiced frames will be included in the average CPP calculation, which might be a very small percentage of the speech. The CPP in this case could be high, if the nonaphonic voiced segments are periodic but would not represent the speech as a whole. Paradoxically, then, accurate voicing detection can actually lead to a higher-than-expected CPP if the voice contains intermittent aphonia.

Ideally, CPP would be calculated only over frames that were *intended* to be voiced. The CAPE-V fully voiced sentence (“We were away a year ago”) can be used for this purpose. More generally, if frames that were not intended to be voiced (including pauses and voiceless consonants) could be accurately excluded, then utterances with different phonemes could be compared. Aphonic segments, which occur during speech that is intended to have voicing, would be included in the CPP computation and lower the result. Applying this criterion automatically would require very accurate segment-level automatic speech recognition, so it may not currently be realistic. Alternatively, CPP algorithms could require that a certain fraction of the recording be voiced in order to calculate CPP (e.g., if the speech is 95% aphonic, no CPP would be returned). Further research would be needed to identify the appropriate fraction of voicing needed to calculate an accurate CPP.

Notably, the *r* ^2^ values for the /a/ vowel regression model were considerably different for the ADSV-based CPP (*r* ^2^ = .71) and Praat-based CPPS (*r* ^2^ = .56). This discrepancy is likely due in part to a single point from one speaker, whose Praat CPPS was relatively high (11.7 dB) but whose ADSV CPP was substantially lower (3.5 dB). This speaker's /a/ vowel received a high overall severity rating of 88 from the trained listeners. The vowel's phonation is characterized by irregular, widely spaced pulses, which were perceived, in this case, as significant strain (mean CAPE-V rating of 90 from trained listeners) and vocal fry. That phonation pattern is likely to be the cause of the discrepancy between the Praat and ADSV CPP values. The algorithms differ in windowing, smoothing, and other parameter settings, and those differences may be particularly sensitive to some property of this specific phonation pattern. Practically, both the Praat CPPS and ADSV CPP values for this speaker were below the clinical cutoff values, so this speaker would have been categorized as having a voice disorder with either program. Still, this finding suggests a need for future work investigating how various CPP computation methods respond to different voice qualities, particularly nonmodal ones.

#### CPP Interpretation in Context

CPP is just one in a set of objective and subjective measures that have been recommended for use in clinical voice assessment and, as such, should be considered/interpreted in the context of the other recommended measures, which include additional acoustic parameters and aerodynamic assessment, as well as subjective listener ratings, medical exam findings (including laryngeal endoscopic imaging), and patient self-report (Patel et al., 2018). In this study, we identified thresholds below which CPP values were associated with the presence of a voice disorder. However, it is conceivable that some voice disorders may lead to abnormally high CPP (e.g., some manifestations of vocal hyperfunction), which a single threshold would not take into account. Recent work on this topic by Awan and Awan (2020) has indicated that rough voices with a strong subharmonic component may exhibit high CPP values and may benefit from a two-stage analysis method that considers the relative heights of cepstral peaks in high and low quefrency ranges. Future work could additionally determine whether it is necessary or possible to also establish upper boundaries for the clinical application of CPP.

In addition to the computation software and speech task, CPP values may be affected by vowel quality, loudness, or a speaker's sex and age. Awan et al. (2012) found that low vowels (e.g., /a/ and /æ/) tended to have higher CPP values than high vowels (e.g., /i/ and /u/) did. Clinicians should ensure that vowel quality is as similar as possible when comparing CPPs based on sustained vowels. Future work could also identify appropriate CPP cutoff values for sustained vowels other than /a/. Additionally, Awan et al. (2012) found that CPP increases significantly with increases in loudness. These increases are likely due to naturally increased glottal closure and reduced perturbation at higher loudness levels and do not reflect changes in underlying dysphonia or voice disorder. Additionally, male speakers tended to have higher CPP than female speakers, possibly because of increased loudness in their normal speaking voices. Similarly, Brockmann-Bauser et al. (2019) found that Praat-based CPPS increased significantly with loudness for both patients with and without voice disorders. Clinicians should therefore use caution when comparing CPP values based on speech samples with different loudness levels.

The patients in the MEEI database ranged in age from 13 to 93 years, whereas the age range of the vocally healthy speakers fell in a smaller bracket (22–59 years). Age is known to often bring voice changes (Mueller, 1997). It may be useful to establish separate normative values for older adults to help distinguish normal aging from disordered voice. This data set did not contain old enough control speakers to establish different norms for older age ranges, but future work could address this question.

## Conclusion

The goal of this study was to employ two frequently used analysis methods to identify values for CPP that can aid clinical voice evaluation, including cutoff thresholds for detecting the presence or absence of a voice disorder and information about how CPP values relate to auditory-perceptual ratings of overall severity of dysphonia. Results from Experiment 1 suggest that ADSV-based CPP values below 11.46 dB (for sustained /a/ vowels) and below 6.11 dB (for the Rainbow Passage) are strongly indicative of the presence of a voice disorder. Corresponding Praat-based CPPS values were 14.45 and 9.33 dB, respectively. Experiment 2 results suggest strong relationships between CPP values and auditory-perceptual ratings of overall severity of dysphonia. Future work could include larger sample sizes to further investigate the relationship between CPP and dysphonia severity, further examination of voicing activity detection in CPP calculation, and investigation into different thresholds for speakers in different age ranges.
